# Prevalence of obesity and smoking among adults in Germany – trends from 2003 to 2023

**DOI:** 10.25646/13038

**Published:** 2025-03-26

**Authors:** Anne Starker, Anja Schienkiewitz, Stefan Damerow, Ronny Kuhnert

**Affiliations:** Robert Koch Institute, Department of Health Monitoring, Berlin, Germany

**Keywords:** Obesity, Smoking, Self-report, Health monitoring, Trends, GEDA, GSTel

## Abstract

**Background:**

Non-communicable diseases (NCDs) are a significant public health challenge. Obesity and smoking are among the major risk factors for their development. In addition to individual behavioural changes, public health measures can help prevent NCDs by creating health-promoting conditions for the population. The aim of this paper is to analyse trends in obesity and smoking prevalence in Germany over time and place these trends in the context of existing public health measures for structural prevention.

**Method:**

The prevalence of obesity and smoking over the last 20 years was examined using data from the Robert Koch Institute Telephone Health Surveys and the German Health Update.

**Results:**

Between 2003 and 2023, the prevalence of obesity increased from 12.2 % to 19.7 % among women and men, for all age and education groups. The prevalence of smoking decreased from 32.1 % to 28.8 %, especially among younger people and in the highly educated group, but the decline has slowed in recent years.

**Conclusions:**

The continuous increase in obesity prevalence between 2003 and 2023 indicates that measures taken so far to prevent obesity have been insufficient. It is therefore imperative not only to address behavioural change at the individual level, but also to implement population-wide, settings-based prevention measures. In addition, more consistent implementation of regulatory measures is needed to further reduce tobacco consumption.

## 1. Introduction

Non-communicable diseases (NCDs) are the leading causes of death worldwide, and their prevalence has been increasingly recognised in recent decades [1, 2]. The most common NCDs include cardiovascular diseases, followed by cancers, chronic respiratory diseases and diabetes mellitus. Globally, these four groups of diseases account for approximately 75 % of all premature NCD deaths [2, 3], affecting not only older adults but also a growing proportion of the younger population. The main modifiable risk factors for NCDs are tobacco use, unhealthy diet, physical inactivity, hazardous alcohol consumption and obesity. For adults in Germany, tobacco use and high body mass index (BMI) are among the most significant risk factors in terms of disease burden and years of life lost due to disability [4, 5]. In Germany in 2019, almost one fifth of adults (19 %) were affected by obesity [6], and more than a quarter (28.9 %) smoked daily or occasionally [7]. This is likely a conservative estimate, as it is based on self-reporting.

According to the World Obesity Atlas 2024, the adult obesity rate in Germany is expected to increase by 0.5 % per year between 2020 and 2035 [8]. This prognosis is based on trends in examination data from 2000 to 2016 and assumes that no behavioural or structural prevention measures are implemented. According to calculations by the Organisation for Economic Co-operation and Development (OECD), Germany is projected to spend around 11 % of its health expenditure on treating obesity-related diseases between 2020 and 2050 [9]. In 2018, the direct costs to the German healthcare system for the treatment of smoking-related diseases and health issues were estimated at 30.3 billion euros [10], or about 15 % of total healthcare spending [11]. When the costs of disability, early retirement and death – the so-called indirect costs (66.9 billion euros) – are included, the total economic costs are estimated at 97.2 billion euros per year [10].

**Key messages**

► Between 2003 and 2023, the prevalence of obesity among adults increased steadily from 12.2 % to 19.7 %, while the prevalence of smoking fell from 32.1 % to 28.8 %.
► The prevalence of obesity has increased in both women and men, and in all age and education groups.
► The decline in smoking prevalence is particularly evident among 18- to 29-year-olds and those with high level of education.
► In Germany, there is currently a lack of health policy concepts for population-wide measures to prevent obesity.
► The reduction in smoking prevalence has slowed in recent years, and Germany is lagging behind in implementing internationally recommended measures.
► Sustainable prevention requires a combination of structural and behavioural measures across all areas of policy.

In 2000, the World Health Organization (WHO) initiated a global strategy for the prevention and control of non-communicable diseases [12]. The Global Action Plan for the Prevention and Control of Non-communicable Diseases 2013 – 2020 [13] and the roadmap for its implementation (2023 – 2030) [14] were launched as follow-up projects. The main objective of these projects is to implement integrated actions to address modifiable risk factors for NCDs and their underlying determinants across different policy areas, following a health in all policies approach. Priority is given to interventions at both the population level (e.g. regulatory measures to promote healthy consumer behaviour through fiscal and market policies) and the individual level (e.g. early detection and treatment of NCDs, including measures to reduce behavioural risk factors). This approach involves a combination of structural and behavioural prevention measures.

Indicators are used to evaluate health improvement interventions and to monitor their successful implementation. These indicators include the proportion of women and men in the population who are affected by obesity or the proportion of current smokers. They are part of the European Core Health Indicators (ECHI), which serve as a basis for comparable monitoring at the European level, and are essential for developing and monitoring strategies and policies to improve population health in Europe [15, 16].

In 2002, Germany first adopted a ‘National Sustainability Strategy’, which since 2016 has been based on the United Nations 2030 Agenda for Sustainable Development with 17 Sustainable Development Goals (SDGs) [17]. Goal 3, ‘Good Health and Well-Being’, aims to ‘ensure healthy lives and promote well-being for all at all ages’. This includes a commitment to halt the rise in obesity rates among adolescents and adults and to reduce the adult smoking rate to 19 %.

The prevention of obesity is challenging due to the complex interplay of individual and structural factors that contribute to its development. Evidence-based approaches exist for the effective prevention of overweight and obesity at the population level. These include the implementation of fiscal interventions, such as the taxation of sugary drinks or the subsidisation of healthy foods. They also include improving access to weight management services through primary health care and efforts to improve dietary and physical activity behaviours across the life course [18]. Obesity prevention measures for adults in Germany are currently mainly limited to individual measures to change diet and promote physical activity. Structural prevention measures targeted at adults, such as the introduction of a tax on foods high in sugar, the reduction of value-added tax (VAT) on fresh, unprocessed foods such as fruit, vegetables and legumes, or the accessible promotion of physical activity programmes, are not currently being pursued in Germany [19]. Population-wide measures, such as clear nutrition labelling on processed products (e.g. NutriScore), are currently voluntary.

The prevention of tobacco use aims both to prevent people from starting smoking and to encourage those who smoke to quit. By signing the 2003 Framework Convention on Tobacco Control (FCTC), an international treaty negotiated under the auspices of the WHO, Germany committed to implementing tobacco control measures proven to be effective in reducing the supply of and demand for tobacco. These include regular increases in tobacco taxes, a comprehensive ban on tobacco advertising, promotion and sponsorship, warnings about the dangers of tobacco use, protection from second-hand smoke, and support for smoking cessation [20]. Since 2002, Germany has implemented several measures to reduce smoking in the population, including increases in tobacco taxes, restrictions on advertising and sales, laws to protect non-smokers and health warnings on tobacco packages (a detailed overview of the measures can be found here [21, 22]).

Cross-sectional surveys that are representative of the population, such as those conducted as part of the health monitoring programme at the Robert Koch Institute (RKI), are suitable for tracking the achievement of the above-mentioned goals using indicators. In addition to reporting on current developments and trends, these surveys can be used to identify the need for action at an early stage, to identify starting points for preventive measures and to evaluate the implementation and success of preventive measures. For example, they can show whether the increase in tobacco product prices, due to the gradual adjustment of tobacco tax rates, influences smoking prevalence, or how the lack of regulatory measures targeted at obesity prevention affects the prevalence of obesity in the population.

The aim of this paper is to show the development of the prevalence of obesity and smoking among adults in Germany over the last 20 years, based on nationwide surveys conducted by RKI, and to place these trends in the context of the above-mentioned public health measures for the prevention of obesity and tobacco use. Estimates are presented for the total population and by sex/gender, age and education group.

## 2. Methods

### 2.1 Data source

The analyses are based on data from the 2003, 2004 and 2006 German Telephone Health Surveys (GSTel) and various waves of the German Health Update (Gesundheit in Deutschland aktuell, GEDA) study conducted by the RKI in 2009, 2010, 2012, 2014/2015, 2019/2020, 2021, 2022 and 2023. With the exception of GEDA 2014/2015-EHIS, all waves of the GEDA study were conducted using computer-assisted telephone interviews (CATI). GEDA 2014/2015-EHIS used a sequential mixed-mode design with self-administered web-based and paper questionnaires. Information on each survey is provided in Annex Table 1. The surveys allow for the identification of trends in the prevalence of obesity and smoking over time among persons aged 18 years and older.

To reduce statistical uncertainty, GSTel 2003 and GSTel 2004 were pooled for the present analyses and are referred to hereinafter as GSTel 2003/2004. After GEDA 2019/2020, a monthly survey of approximately 1,000 persons started in July 2021 (GEDA 2021). As height and weight were only collected for three months in GEDA 2021, the sample size is too small to estimate obesity prevalences stratified by age, sex/gender and education with sufficient statistical precision. Therefore, the values for obesity from 2021 are not included in this analysis. Smoking status was recorded throughout the study period (07 – 12/2021) so that smoking prevalence can be calculated for GEDA 2021. In GEDA 2022 and GEDA 2023, smoking status was collected from 02/2022 to 03/2023 and is summarised as GEDA 2022/2023. Information on height and weight was available at all survey points, so that obesity prevalence estimates are possible for both GEDA 2022 and GEDA 2023.

### 2.2 Instruments and indicators

#### Obesity

The survey questionnaires include self-reported data on body weight and height (Table 1), with slightly different wording of the question but always the same response categories. Body mass index (BMI, kg/m^2^) was calculated from these values. According to the WHO classification [23], a BMI ≥ 30 kg/m^2^ is defined as obesity.

#### Smoking status

Smoking status (of tobacco products) was based on self-reports, with slightly different wording of the question but always the same response categories (Table 1). The response categories can be used to show the prevalence of current smoking (yes, daily or yes, occasionally).

### 2.3 Statistical Methods

The analyses are differentiated by the time of study, based on weighted prevalences with 95 % confidence intervals. To allow for comparison of the prevalence estimates, direct age standardisation was used in the calculation. The age structures of the samples were adjusted to the standard European population for the year 2013 [24]. The data are disaggregated by sex/gender (female, male), age (18 – 29, 30 – 44, 45 – 64, ≥ 65 years) and education groups (low, medium, high) according to the CASMIN classification (Comparative Analyses of Social Mobility in Industrial Nations) [25].

The calculation of the sample weights is study-specific and accounts for the different participation probabilities within the sample design (Annex Table 1). With the exception of GEDA 2014/2015-EHIS, the selection frame is a landline sample until GEDA 2012 and a mobile and landline sample from GEDA 2019/2020 onwards. GEDA 2014/2015-EHIS is a two-stage clustered sample from official residency registries and accounts for both the selection probability of the survey locations and the selection probability of the participants within the locations when calculating the probability of participation.

To adjust for individual willingness to participate, the net samples of each survey are adjusted based on population data from the Federal Statistical Office and the German microcensus corresponding to the respective study years. In this context, age and sex distributions (specific to each federal state) are taken into account, as well as the type of district and the distribution of education according to the International Standard Classification of Education (ISCED) [26]. The levels of adjustment are described in the respective methodological publications, with the exception of GSTel04 and GSTel06 (Annex Table 1). For GSTel04 and GSTel06, the regional levels (GStel04: Federal State; GStel06: North, North Rhine-Westphalia, Central, East, Bavaria, Baden-Württemberg) were adjusted by sex x age groups (18 – 24, 25 – 29, …, 65 – 69, 70 years and older) and sex x age groups (18 – 24, 25 – 29, 30 – 39, …, 60 – 69, 70 years and older) x education (ISCED97 categories: low, medium, high).

The trend between GSTel03 and GEDA 2022/2023 and GEDA 2023 is tested using a univariate logistic regression. A difference is considered statistically significant if the *p*-value, when accounting for the weighting and survey design, is less than 0.05.

There may be deviations from previously published results for obesity and smoking prevalence in Germany. This is due to the application of direct age-standardisation in the present study and, in some cases, adjustments to the weighting factors to reflect the regional and educational distribution in the population in a comparable way across all data sets.

All results were calculated using the statistical software R (version 4.3.0) with the packages ‘survey’ and ‘srvyr’.

## 3. Results

### 3.1 Obesity

**Figure 1:**
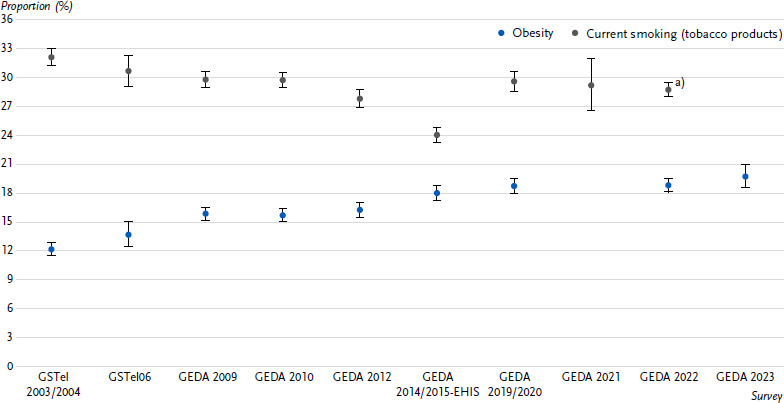
Weighted age-standardised prevalence for obesity and current smoking (proportions with 95 % confidence interval). Source: Nationwide Health Interview Surveys of the Robert Koch Institute 2003 – 2023 a) Smoking status was assessed in the period 02/2022 to 03/2023 (t1 – t13) and is referred to as GEDA 2022/2023

Between GSTel 2003/2004 and GEDA 2009, the percentage of women and men with obesity increased from 12.2 % to 15.9 % (Figure 1). Between GEDA 2010 and GEDA 2023, this percentage continued to increase steadily from 15.7 % to 19.7 %. Irrespective of changes in the composition of the population, the increase in obesity prevalence between 2003 and 2023 is statistically significant (*p* < 0.0001).

This trend applies to both women and men (Table 2), although the prevalence estimates for women are slightly, but not statistically significantly, lower than for men. The increase in obesity prevalence is evident across all age and education groups. Among 18- to 29-year-olds, obesity prevalence increased from 3.4 % in 2003/2004 to 11.3 % in 2023, among 30- to 44-year-olds from 9.2 % to 19.2 %, among 45- to 64-year-olds from 16.9 % to 24.2 % and among people aged 65 years and older from 15.3 % to 20.5 %. In the low education group, obesity prevalence increased from 16.0 % to 28.7 %, in the medium education group from 10.4 % to 19.4 % and in the high education group from 6.4 % to 11.3 %.

### 3.2 Smoking

Overall, the proportion of current smokers in the population decreased from 32.1 % in 2003 to 28.8 % in 2022/2023 (Figure 1). There is a notable decrease in smoking prevalence from GEDA 2012 to GEDA 2014/2015-EHIS, followed by an increase from GEDA 2014/2015-EHIS to GEDA 2019/2020, which is above the level of GEDA 2012. However, the decline in smoking prevalence has slowed in recent years and, with the exception of GEDA 2014/2015-EHIS, has not changed significantly since 2006. These trends are similar for both women and men (Table 2), although smoking prevalence is significantly lower among women than men. This difference (around eight percentage points) hardly changes over the entire observation period. With regard to age, only the two younger age groups show a decrease. Among 18- to 29-year-olds, the proportion of current smokers fell from 47.5 % to 35.4 %, and among 30- to 44-year-olds from 41.2 % to 33.6 %. In terms of education, the decline in the proportion of smokers is particularly marked in the high education group (21.3 % to 16.9 %). Overall, educational differences in smoking prevalence have increased over time.

## 4. Discussion

### 4.1 Main results

From 2003 to 2023, the prevalence of obesity in the total population has steadily increased, while the prevalence of current smoking has decreased, with some fluctuations and a slowed decline in recent years. The increase in the prevalence of obesity is observed in both women and men and in all age and education groups. The decrease in the prevalence of current smoking is also observed in both women and men, although differences exist by age and education, with younger age groups and those with higher education showing more pronounced declines.

### 4.2 Contextualisation and interpretation

#### Obesity trend results

The increase in the prevalence of obesity in adults over the last 20 years, which has been observed in both women and men and across all age and education groups, has been documented both nationally [27, 28] and internationally [29, 30] in survey and examination data that include information on height and weight. In Germany, the microcensus shows an increase of 4.7 percentage points between 1999 and 2021, with obesity prevalence increasing from 10.7 % in 1999 to 15.4 % in 2021 [28]. Data from the Socio-Economic Panel (SOEP) also show a significant increase in the predicted probability of obesity between 2002 and 2020, irrespective of sex, age and education group. There is an apparent increase in educational inequality in obesity prevalence in terms of the size of the difference between the highest and lowest education groups [27]. It is also clear that between GSTel 2003/2004 and GEDA 2023, the highest obesity prevalence is found in the lowest education groups at all survey times; a fact that has already been described in previous trend analyses of national health surveys [31]. Although the absolute prevalence is not identical between the different data sources, the order of magnitude of the estimates are comparable and the trend over time is consistent.

The prevalence trend presented here from the GSTel and GEDA is based on self-reports by the respondents and thus differs from estimates based on measurement data from interview and examination surveys, such as the East-West Survey 1990 – 1992, the German Health Interview and Examination Survey 1998 (GNHIES98) and the German Health Interview and Examination Survey for Adults (DEGS, 2008 – 2011) [32, 33]. Based on DEGS 2008 – 2011 survey, the prevalence of obesity was 23.9 % for women and 23.3 % for men, significantly higher than the prevalences presented here. When directly comparing trends from different surveys, it should be noted that prevalences calculated from self-reports are lower (see Strengths and limitations). Furthermore, not only the mode of data collection (survey data vs. measurement data) but also the sample access method (official residency registry vs. telephone) and the instrument (questionnaire vs. telephone interview) should be comparable for each survey wave. It can therefore be assumed that the prevalence of obesity in Germany is likely conservatively estimated in the present study.

#### Results in the context of health policy measures

According to the WHO, halting the rise in obesity is critical to achieving the Sustainable Development Goal (SDG 3.4) of reducing premature mortality from NCDs by 2030 [19]. There is currently no country in the world that has implemented comprehensive measures to halt the rise in obesity prevalence. This also applies to Germany. According to the WHO, action at the individual and societal level is needed to reduce the development of NCDs associated with obesity. To address the complexity of the obesity epidemic, the WHO recommends focusing on multi-sectoral interventions, such as food production, marketing and pricing, and interventions that address health determinants, such as poverty reduction and urban planning [19]. Prevention measures that focus solely on individuals and their behaviour, without taking into account structural and socioeconomic factors, will not be effective enough [34], and a further increase in the prevalence of obesity can be expected. In Germany, there are currently calls for individuals to prioritise a healthy and sustainable diet and sufficient physical activity in their daily lives [35]. However, there is no tax on foods with a high sugar content, nor is there a reduced VAT rate for unprocessed foods. The implementation of tax increases on unhealthy products, a strategy that has been shown to be effective for tobacco products and to reduce consumption [36], is not currently under discussion for highly processed foods. A significant proportion of highly processed foods are energy-dense, nutrient-poor and low cost [37], which makes them easy to consume in everyday life. However, there is currently insufficient information on their possible health consequences [38, 39]. One of the few legislative measures aimed at preventing obesity in the broadest sense is the NutriScore, which is intended to enable the population to compare the nutritional composition of foods within a product group and choose options with a more favorable nutritional label. However, the use of NutriScore is voluntary [40]. In addition to shifts in dietary habits toward highly processed and calorie-dense foods, a living environment dominated by motorised transport and sedentary behaviours has contributed to a pattern of physical inactivity. Changes to the living environment are needed to encourage more outdoor activities and create more opportunities for physical activity in everyday life by improving access to sports facilities, in order to increase physical activity and get people moving [41].

When obesity is present, long-term, appropriate and comprehensive management of this disease is required. A structured disease management programme (DMP) for patients with obesity is currently implemented [42]. While treatments for obesity, such as the use of weight-loss drugs or gastric bypass surgery, are important and must be available to patients, population-based preventive measures, as addressed in the current guideline on the ‘Prevention and Treatment of Obesity’ [43], should not be neglected by science and policy.

#### Smoking trend results

The decline in smoking prevalence among adults is also shown by other population-based studies, such as the Epidemiological Addiction Survey (ESA), with data at three-year intervals from 1995 to 2021 for the population aged between 18 to 59 years [44], and the German microcensus, with data at four-year intervals from 2003 to 2017 for the population aged 15 years and older [45]. The prevalences in these surveys are similar to those in the present analysis, although comparability is limited due to methodological differences in sampling, data collection methods and the wording of survey questions. In contrast to the GEDA waves, the results of the ESA and the microcensus show a continuous decline in smoking prevalence. However, in both studies there was no change in the wording of the question and also no change in the method of access to the sample (official residency registry vs. telephone) as in GEDA (Table 1). It should also be noted that the survey duration of each wave of the ESA and the microcensus differs from that of the RKI surveys examined. The smoking prevalence in GEDA 2019/2020 refers to a period of 19 months (Annex Table 1), which includes different phases of the COVID-19 pandemic. However, no pandemic-related changes in tobacco smoking prevalence were observed in the GEDA 2019/2020 data [46]. This is in contrast to the results of the German Smoking Behaviour Survey (DEBRA) [47]. The DEBRA study has been investigating the use of tobacco and alternative nicotine delivery systems in the German population aged 14 years and older since 2016.

Methodological reasons for the deviation in smoking prevalence between GEDA waves cannot be excluded (see Strengths and limitations).

The results also show that the trends are continuing, with fewer younger people smoking, educational differences in smoking prevalence persisting, and smoking rates still higher in men than in women, as confirmed by previous trend analyses [48–50].

#### Results in the context of health policy measures

The regulatory measures taken in Germany since 2002 to reduce tobacco consumption in the population and to protect non-smokers from the health hazards of second-hand smoke have been accompanied by the national health target ‘Reduce tobacco consumption’, which was initiated in 2003 and updated in 2015 [51]. Health targets are a complementary steering instrument in the health care system that pursue a health in all policies approach [52]. For the target ‘Reduce tobacco consumption’, the following objectives were defined: adolescents and young adults remain non-smokers, smoking cessation is increased in all age groups, and comprehensive protection from passive smoking is guaranteed [51]. Because of its importance for health policy, this health target was also included in the Prevention Act 2015 [53]. This means that the content relevant to this health target can be considered in the ‘Prevention Guidelines’ of statutory health insurance, which define the fields of action and criteria for the services provided by health insurance companies for primary prevention and health promotion.

The decline in smoking prevalence can, therefore, be considered a success of the implemented tobacco control measures [54, 55], although the exact effects of individual measures are difficult to quantify. Internationally, the effectiveness of the measures recommended by the FCTC has been confirmed [56]. Nevertheless, Germany still has work to do in implementing the internationally recommended tobacco control measures, as reflected in the European Tobacco Control Scale 2021. This scale compares 37 countries in terms of their efforts to effectively prevent and control tobacco use. Germany ranks fourth from the bottom, with the United Kingdom and Ireland at the top [57]. These two countries are well ahead of Germany, particularly in terms of consistent and comprehensive protection of non-smokers, continuous increases in tobacco taxes and comprehensive cessation services [58].

One of the most effective measures to discourage children and adolescents from smoking is a significant and continuous increase in tobacco taxes [59]. This is because children and adolescents are more sensitive to price increases than adults, as they have less disposable income. The development of smoking behaviour among young people in Germany confirms this: the proportion of smokers among 12- to 17-year-olds fell significantly after the substantial tax increases from 2002 to 2005 [60]. The continued decline in the proportion of young people who smoke in Germany can also be explained by other tobacco control measures, although these are estimated to have a much smaller reach than tax regulations [54]. As fewer young people take up smoking, this trend continues into the young adult age group, leading to an overall decline in smoking prevalence.

Tobacco control interventions do not explicitly address social inequalities [61], and evidence suggests that the effectiveness of these interventions varies across education and socioeconomic groups [62, 63]. Specific measures and interventions should therefore be targeted to reach smokers with lower levels of education. However, it has also been shown that all population groups, including the socially disadvantaged, benefit from the continuous and consistent implementation of population-level tobacco control measures, and that this can contribute to reducing health inequalities [64].

It should also be noted that smoking prevalence in Germany has been declining since the 1990s, even before tobacco control measures were introduced [33, 65]. Therefore, trends in tobacco consumption should be viewed in the context of broader societal changes, such as the growing awareness of the health consequences of smoking and changing social norms. For these reasons, it is important to continue collecting data on the extent, patterns, determinants and consequences of tobacco consumption and exposure, as required by the FCTC, as well as data on other products, such as e-cigarettes and heat-not-burn devices, and to monitor trends over time.

### 4.3 Strengths and limitations

For the present findings, data from surveys conducted over the last 20 years were analysed independently of changes in the age structure of the population, which allow statements to be made about trends in obesity and current smoking among adults over time. Trends for women and men in different age and education groups are presented and placed in the context of public health measures to prevent obesity and smoking.

Educational inequalities in obesity prevalence have been described using age-standardised prevalence differences, which is a common way of presenting educational inequalities between different groups. As trends in health inequalities can depend significantly on whether relative or absolute differences are considered, further analysis of absolute and relative educational differences are required by calculating the Slope Index of Inequality (SII) and the Relative Index of Inequality (RII) [49].

Another limitation is the use of self-reported body weight and height, which can be biased: weight is often underestimated compared with standardised measurements, while height tends to be overestimated, resulting in a BMI that is lower than a BMI determined by measurement [66]. Thus, the prevalence of obesity based on self-reports is lower than that based on measurement data, and the current extent of obesity may be significantly underestimated. An increase in the prevalence of obesity has already been observed in analyses of examination data with time series from 1990 – 1992 to 2008 – 2011 [32, 33]. The data on tobacco use are also based on self-reports. The results could be biased by socially desirable response behaviour, leading to an underestimation of the prevalence of current smoking. As smoking becomes less socially acceptable over time, this bias may lead to an over-estimation of the decline in the trend. The change in question wording described above limits comparability over time. In addition, e-cigarette use was not included in this analysis. However, it is likely that some smokers have switched to using e-cigarettes in recent years or are using these products in combination with conventional tobacco cigarettes. Figures from the DEBRA study show that e-cigarette use has increased in the population, particularly since 2022 [67]. This suggests that overall nicotine consumption – from tobacco and e-cigarettes – may have increased. This trend is also being observed in England [68, 69]. However, there has not yet been a systematic evaluation of tobacco control policies in Germany.

A limitation when comparing surveys over time is the mode of data collection used in GEDA 2014/2015-EHIS, which differs from other GEDA waves and the Telephone Health Surveys. GEDA 2014/2015-EHIS used a sequential mixed-mode design, where participants were first invited to participate online and those who did not respond within four weeks were sent a paper questionnaire by mail. Due to the lower dispersion of weights in the GEDA 2014/2015-EHIS, it is also assumed that the two-stage stratified cluster sample, randomly selected from official residency registries, better represents the population in Germany than the GEDA telephone surveys. Although the weighting adjusts for known selection mechanisms, there may still be biases. For example, participants in the GEDA telephone survey require both hearing ability and knowledge of the German language.

Despite the methodological limitations described, the present article allows a critical assessment of the results on smoking prevalence in the context of health policy measures to reduce tobacco consumption. The same applies to population-level measures to prevent obesity.

### 4.4 Conclusions

When the major NCD risk factors of obesity and smoking are considered together, opposing trends emerge. This could be due to different population-based prevention strategies being applied to each risk factor. The complexity of these risk factors varies greatly in how they interact with individual behaviours and lifestyle factors.

The continuous increase in the prevalence of obesity between 2003 and 2023 shows that previous efforts to prevent obesity have been insufficient. In Germany, there are currently no effective or binding population-wide approaches to prevent obesity. The effects of individual behavioural prevention are considered to be limited. To achieve a sustainable effect on obesity prevention, population-wide measures are necessary.

In the case of smoking, a combination of regulatory measures (such as higher taxes on tobacco products and smoking bans in public places) and individual measures (such as smoking cessation therapies and medication) have been implemented over the last 20 years. However, there are no new tobacco control measures planned in Germany, so it can be assumed that there will be no change in the country’s ranking on the Tobacco Control Scale or in smoking prevalence in the foreseeable future [70, 71]. Germany lags behind other countries in the consistent implementation of tobacco control measures, and no long-term strategy exists to ensure a sustainable reduction in tobacco consumption. Because of this, a broad alliance of health organisations has proposed ten specific measures based on the FCTC, together with a binding roadmap for policymakers, to achieve a tobacco-free Germany by 2040 [72]. However, policymakers have not yet taken up these measures.

Monitoring key risk factors is essential for understanding their prevalence in the population and assessing the impact of population-wide behavioural and structural prevention strategies. The newly established German Health Interview and Examination Survey (Gesundheit in Deutschland) [73] could help address questions about the barriers and drivers of individual behaviour (WHO’s Behavioural and Cultural Insights (BCI) approach). However, in the context of structural prevention, it is also necessary to pursue a health in all policies approach, as a combination of structural and behavioural prevention is likely to be successful [19, 72]. Ongoing monitoring of the obesity and smoking prevalence makes it possible to assess the effectiveness of prevention measures and track progress toward national targets [73].

## Figures and Tables

**Table 1:** Assessment of height, weight and smoking status in the Nationwide Health Interview Surveys of the Robert Koch Institute

Survey	Question	Possible answers
**Body height**		
GSTel03, GSTel04, GSTel06	How tall are you – in cm?	Value Don‘t know/not sure No response/refusal
GEDA 2009	How tall are you – in cm?	Value Don‘t know No response
GEDA 2010, GEDA 2012	How tall are you (without shoes) – in cm?	
GEDA 2014/2015-EHIS	How tall are you without shoes? Please state your height in cm.	Value
GEDA 2019/2020, GEDA 2021, GEDA 2022, GEDA 2023	How tall are you without shoes? Please state your height in cm.	Value Don‘t know No response
**Body weight**		
GSTel03, GSTel04, GSTel06	How much do you weigh – in kg?	Value Don‘t know/not sure No response/refusal
GEDA 2009	How much do you weigh – in kg?	Value Don‘t know No response
GEDA 2010, GEDA 2012	How much do you weigh (without clothes) – in kg?	
GEDA 2014/2015-EHIS	How much do you weigh without clothes and shoes? Please state your body weight in kg. Interviewer‘s note: Pregnant women, please state your pre-pregnancy weight.	Value
GEDA 2019/2020, GEDA 2021, GEDA 2022, GEDA 2023	How much do you weigh without clothes and shoes? Please state your body weight in kg. Interviewer‘s note: Pregnant women, please state your pre-pregnancy weight.	Value Don‘t know No response
**Smoking**		
GSTel03, GSTel04, GSTel06, GEDA 2009, GEDA 2010, GEDA 2012	Do you currently smoke, even if only occasionally?	Yes, daily Yes, occasionally No, not any more Never smoked Don‘t know No response
GEDA 2014/2015-EHIS	Do you smoke?	
GEDA 2019/2020, GEDA 2021, GEDA 2022, GEDA 2023	Do you smoke any tobacco products, including heated tobacco products? Please exclude electronic cigarettes or similar devices. Interviewer‘s note: Heated tobacco products refer to products such as IQOS or HEETS tobacco sticks.	

**Table 2:** Weighted age-standardised prevalences for obesity and current smoking (proportions with 95 % confidence interval). Source: Nationwide Health Interview Surveys of the Robert Koch Institute 2003 – 2023

Survey	GSTel 2003/2004^a)^		GSTel06		GEDA 2009		GEDA 2010		GEDA 2012		GEDA 2014/2015-EHIS		GEDA 2019/2020^b)^		GEDA 2022		GEDA 2023	
	%	(95 % CI)	%	(95 % CI)	%	(95 % CI)	%	(95 % CI)	%	(95 % CI)	%	(95 % CI)	%	(95 % CI)	%	(95 % CI)	%	(95 % CI)
**Obesity**																		
**Total**	**12.2**	**(11.5 – 12.9)**	**13.7**	**(12.5 – 15.0)**	**15.9**	**(15.1 – 16.6)**	**15.7**	**(15.0 – 16.4)**	**16.3**	**(15.5 – 17.1)**	**18.0**	**(17.3 – 18.8)**	**18.8**	**(18.0 – 19.6)**	**18.8**	**(18.1 – 19.6)**	**19.7**	**(18.6 – 21.0)**
**Sex/gender**																		
Women	12.0	(11.1 – 12.9)	12.8	(11.2 – 14.6)	15.3	(14.4 – 16.3)	15.4	(14.5 – 16.4)	15.8	(14.8 – 17.0)	17.8	(16.9 – 18.7)	18.5	(17.4 – 19.7)	18.4	(17.4 – 19.4)	18.2	(16.7 – 19.9)
Men	12.4	(11.4 – 13.4)	14.6	(12.8 – 16.6)	16.3	(15.3 – 17.4)	16.0	(15.0 – 17.0)	16.7	(15.6 – 17.9)	18.3	(17.3 – 19.4)	19.0	(17.8 – 20.2)	19.3	(18.3 – 20.3)	21.2	(19.4 – 23.1)
**Age group**																		
18 – 29 years	3.4	(2.7 – 4.2)	6.7	(4.9 – 9.1)	6.3	(5.3 – 7.4)	5.7	(4.8 – 6.7)	7.0	(5.7 – 8.5)	9.5	(8.3 – 10.7)	9.7	(8.0 – 11.7)	11.3	(9.7 – 13.1)	11.3	(8.7 – 14.6)
30 – 44 years	9.2	(8.4 – 10.1)	11.2	(9.4 – 13.2)	12.1	(11.0 – 13.3)	14.4	(13.2 – 15.7)	14.4	(12.8 – 16.1)	17.4	(16.0 – 19.0)	16.8	(15.1 – 18.6)	18.5	(16.9 – 20.2)	19.2	(16.5 – 22.1)
45 – 64 years	16.9	(15.6 – 18.2)	17.6	(15.4 – 20.1)	20.3	(19.0 – 21.6)	20.0	(18.8 – 21.3)	19.7	(18.3 – 21.1)	20.9	(19.8 – 22.0)	23.2	(21.8 – 24.7)	22.8	(21.6 – 24.1)	24.2	(22.2 – 26.4)
≥ 65 years	15.3	(13.5 – 17.4)	16.2	(13.0 – 19.9)	20.7	(18.9 – 22.6)	18.6	(16.9 – 20.4)	20.4	(18.7 – 22.2)	21.0	(19.7 – 22.5)	21.4	(19.9 – 23.0)	19.3	(18.3 – 20.4)	20.5	(18.7 – 22.4)
**Education group**																		
Low	16.0	(14.8 – 17.4)	18.5	(15.9 – 21.4)	20.4	(19.1 – 21.9)	20.3	(18.9 – 21.7)	20.9	(19.1 – 22.9)	23.9	(22.3 – 25.7)	26.2	(23.9 – 28.5)	25.7	(23.4 – 28.1)	28.7	(25.0 – 32.7)
Medium	10.4	(9.4 – 11.6)	11.5	(9.9 – 13.3)	14.3	(13.3 – 15.4)	13.8	(12.9 – 14.8)	14.7	(13.7 – 15.8)	17.4	(16.5 – 18.3)	17.7	(16.6 – 18.8)	18.6	(17.7 – 19.6)	19.4	(17.8 – 21.0)
High	6.4	(5.5 – 7.4)	9.8	(7.9 – 12.2)	8.5	(7.6 – 9.4)	9.0	(8.2 – 9.9)	9.9	(9.0 – 10.9)	10.1	(9.2 – 11.2)	10.4	(9.7 – 11.2)	11.1	(10.4 – 11.9)	11.3	(10.2 – 12.5)
																		
**Survey**	**GSTel 2003/2004^a)^**		**GSTel06**		**GEDA 2009**		**GEDA 2010**		**GEDA 2012**		**GEDA 2014/2015-EHIS**		**GEDA 2019/2020^b)^**		**GEDA 2021**		**GEDA 2022/2023^c)^**	
	**%**	**(95 % CI)**	**%**	**(95 % CI)**	**%**	**(95 % CI)**	**%**	**(95 % CI)**	**%**	**(95 % CI)**	**%**	**(95 % CI)**	**%**	**(95 % CI)**	**%**	**(95 % CI)**	**%**	**(95 % CI)**
**Smoking**																		
**Total**	**32.1**	**(31.3 – 33.0)**	**30.7**	**(29.1 – 32.4)**	**29.8**	**(29.0 – 30.6)**	**29.8**	**(29.0 – 30.6)**	**27.8**	**(26.9 – 28.8)**	**24.1**	**(23.3 – 24.8)**	**29.6**	**(28.6 – 30.7)**	**29.2**	**(26.6 – 32.0)**	**28.8**	**(28.0 – 29.5)**
**Sex/gender**																		
Women	28.3	(27.2 – 29.4)	27.9	(25.9 – 30.0)	26.7	(25.7 – 27.7)	26.9	(25.9 – 27.9)	24.8	(23.6 – 26.0)	21.4	(20.5 – 22.4)	25.1	(23.7 – 26.4)	25.9	(22.3 – 29.8)	24.8	(23.8 – 25.8)
Men	36.0	(34.7 – 37.4)	33.5	(31.0 – 36.1)	32.9	(31.7 – 34.2)	32.6	(31.4 – 33.8)	30.9	(29.6 – 32.3)	26.7	(25.6 – 27.8)	34.2	(32.7 – 35.7)	32.5	(28.7 – 36.6)	32.7	(31.6 – 33.9)
**Age group**																		
18 – 29 years	47.5	(45.4 – 49.6)	41.8	(38.3 – 45.4)	41.4	(39.5 – 43.3)	39.8	(37.9 – 41.7)	35.1	(32.8 – 37.4)	32.0	(30.0 – 34.0)	35.5	(32.8 – 38.4)	26.1	(19.6 – 33.8)	35.4	(33.2 – 37.6)
30 – 44 years	41.2	(39.7 – 42.6)	41.5	(38.8 – 44.4)	38.0	(36.3 – 39.6)	39.5	(37.9 – 41.1)	35.4	(33.4 – 37.4)	31.2	(29.6 – 32.9)	37.3	(35.0 – 39.7)	36.7	(30.9 – 42.9)	33.6	(31.9 – 35.3)
45 – 64 years	31.9	(30.4 – 33.4)	32.4	(29.7 – 35.3)	31.4	(30.1 – 32.9)	31.2	(29.8 – 32.5)	30.0	(28.5 – 31.5)	26.0	(24.9 – 27.2)	32.5	(30.9 – 34.1)	35.8	(31.4 – 40.5)	30.9	(29.7 – 32.1)
≥ 65 years	11.8	(10.2 – 13.7)	8.9	(6.8 – 11.5)	10.6	(9.4 – 12.1)	10.4	(9.1 – 11.8)	11.8	(10.4 – 13.3)	8.0	(7.3 – 8.8)	13.4	(12.0 – 14.8)	13.9	(10.9 – 17.6)	16.0	(15.1 – 17.1)
**Education group**																		
Low	40.3	(38.5 – 42.1)	35.9	(32.6 – 39.3)	34.9	(33.3 – 36.7)	36.8	(35.1 – 38.5)	34.7	(32.4 – 37.0)	30.6	(28.6 – 32.6)	38.9	(36.3 – 41.6)	35.1	(27.2 – 43.9)	37.7	(35.5 – 39.9)
Medium	32.3	(30.9 – 33.8)	31.1	(28.7 – 33.6)	30.2	(29.0 – 31.4)	29.8	(28.6 – 31.0)	28.1	(26.9 – 29.4)	24.5	(23.6 – 25.5)	30.5	(29.2 – 31.9)	32.5	(28.9 – 36.4)	30.0	(29.0 – 31.0)
High	21.3	(19.5 – 23.3)	24.1	(21.1 – 27.4)	19.4	(18.0 – 20.8)	18.9	(17.7 – 20.2)	18.3	(16.9 – 19.7)	15.7	(14.5 – 16.9)	17.0	(15.9 – 18.2)	15.4	(12.8 – 18.5)	16.9	(16.1 – 17.8)

a) Pooled dataset for GSTel03 and GSTel04

b) Extension of GEDA 2019/2020-EHIS

c) Smoking status was assessed in the period 02/2022 to 03/2023 (t1 – t13) and is referred to as GEDA 2022/2023

95 % CI = 95 % confidence interval

## Appendix

**Annex Table 1:** Overview of the German Telephone Health Surveys and the different waves of the study German Health Update 2009 to 2023

Acronym	Survey period	Sample	Sample design	Survey mode	Data record version	Reference
GSTel03	09/2002 to 03/2003	Total: 8,318 Women: 4,446 Men: 3,872	Randomised last digits procedure of a telephone number sample created according to the Gabler-Häder design	CATI	V6	[74, 75]
GSTel04	09/2003 to 04/2004	Total: 7,341 Women: 3,965 Men: 3,376	Randomised last digits procedure of a telephone number sample created according to the Gabler-Häder design	CATI	V2	[76]
GSTel06	10/2005 to 03/2006	Total: 5,542 Women: 3,066 Men: 2,476	Basis was a gross sample of 40,720 dialled numbers generated according to the Gabler-Häder method, provided by ZUMA Mannheim	CATI	V3	[77]
GEDA 2009	07/2008 to 05/2009	Total: 21,262 Women: 12,114 Men: 9,148	Germany-wide representative random sample based on a randomly generated telephone sample	CATI	V22	[78]
GEDA 2010	09/2009 to 07/2010	Total: 22,050 Women: 12,483 Men: 9,567		CATI	V9	[79]
GEDA 2012	03/2012 to 03/2023	Total: 19,294 Women: 9,976 Men: 9,318	Germany-wide representative random sample from an ADM landline sampling system	CATI	V5	[80]
GEDA 2014/2015-EHIS	11/2014 to 07/2015	Total: 24,016 Women: 13,144 Men: 10,872	Two-stage stratified cluster sample randomly drawn from official residency registries, parent population is the population aged 15 years and over with permanent residence in Germany	standardised web-based or paper-based questionnaire (sequential mixed-mode design)	V7	[81]
GEDA 2019/2020^*^	04/2019 to 01/2021	Total: 26,507 Women: 11,968 Men: 10,740	German-wide representative dual-frame telephone sample system (mobile and landline)	CATI	V4	[82]
GEDA 2021	6/2021 to 12/2021	Total: 4,971 Women: 2,587 Men: 2,384		CATI	V2	https://www.rki.de/EN/Topics/Noncommunicable-diseases/Health-surveys/Studies/gedagerman-health-update.html?nn=16782096
GEDA 2022	02/2022 to 01/2023	Total: 33,149 Women: 18,029 Men: 15,120		CATI	V3	
GEDA 2023	01/2023 to 02/2024	Total: 30,002 Women: 16,108 Men: 13,894		CATI	V1	

*GEDA 2019/2020 is an extension of the GEDA 2019/2020-EHIS study [82] covering the period from September 2020 to January 2021

CATI = Computer-assisted telephone interview, ADM = Arbeitskreis Deutscher Markt- und Sozialforschungsinstitute e.V.
